# Culture-Negative Native Vertebral Osteomyelitis: A Narrative Review of an Underdescribed Condition

**DOI:** 10.3390/jcm13195802

**Published:** 2024-09-28

**Authors:** Seyed Mohammad Amin Alavi, Francesco Petri, Omar K. Mahmoud, Rita Igwilo-Alaneme, Said El Zein, Ahmad N. Nassr, Andrea Gori, Elie F. Berbari

**Affiliations:** 1 Faculty of Medicine, Ahvaz Jundishapur University of Medical Sciences, Ahvaz 6135715794, Iran; sma.alavi94@gmail.com; 2 Division of Public Health, Infectious Diseases and Occupational Medicine, Department of Medicine, Mayo Clinic College of Medicine and Science, Mayo Clinic, Rochester, MN 55905, USA; francescopetri2@gmail.com (F.P.); omarmhmud1@gmail.com (O.K.M.); igwilo-alaneme.rita@mayo.edu (R.I.-A.); selzein20@gmail.com (S.E.Z.); 3 Department of Infectious Diseases, ASST Fatebenefratelli Sacco, “L. Sacco” University Hospital, 20157 Milan, Italy; andrea.gori@unimi.it; 4 Department of Orthopedic Surgery, Mayo Clinic, Rochester, MN 55905, USA; nassr.ahmad@mayo.edu; 5 Centre of Multidisciplinary Research in Health Science (MACH), University of Milan, 20122 Milan, Italy

**Keywords:** discitis, diagnosis, management, negative culture, spondylitis, vertebral osteomyelitis

## Abstract

The incidence of culture-negative NVO (CN-NVO) cases is increasing, presenting significant diagnostic and therapeutic challenges due to the inability to isolate causative organisms with conventional microbiological methods. Factors influencing the diagnosis of CN-NVO include prior antimicrobial therapy, low pathogen burden, fastidious or intracellular organisms, technical issues, and non-infectious mimickers. Diagnosis often relies on imaging modalities like magnetic resonance imaging (MRI) and computed tomography (CT)-guided biopsy, though these methods can sometimes fail to yield positive microbiological results. Advanced diagnostic tools, such as polymerase chain reaction (PCR), metagenomic next-generation sequencing (mNGS), and cell-free DNA analysis, may be necessary to identify the pathogen. The causative pathogen cannot be isolated in some patients, among which an empirical antimicrobial therapy should be initiated. This narrative review discusses the management, monitoring, surgical indications, and outcomes for patients with CN-NVO.

## 1. Introduction

Native vertebral osteomyelitis (NVO), also known as spondylodiscitis, spondylitis, or discitis, is a rare condition that accounts for 3–5% of all cases of osteomyelitis [1,2]. The hospital admission rate among patients with NVO was 4.8 per 100,000, with an overall increase in recent years from 2.9 to 5.4 per 100,000 patients, and is associated with a significant mortality rate of up to 20% [3,4]. This increase in incidence has been attributed to improved diagnostic methods, compounded with the growing prevalence of risk factors such as advanced age and immunosuppression [5].

Accurately identifying microorganisms is essential for determining the optimal antimicrobial treatment for NVO [6]. Nevertheless, the microbiological etiology cannot be identified in up to 37–50% of patients with suspected NVO despite using several techniques such as blood cultures, serology, surgical or image-guided aspiration biopsy, and, more recently, molecular methods [7,8,9,10,11]. Over the period spanning 1995–2008, a parallel increase in the incidence of culture-negative NVO (CN-NVO) from 0.3 to 1.8 cases per 100,000 people has been described [12].

Choosing an appropriate antimicrobial agent for patients diagnosed with CN-NVO is challenging as it is based on empirical therapy and clinical suspicion, together with epidemiological awareness rather than organism-specific criteria [13], and because of the lack of agreed-upon antimicrobial regimens for treating CN-NVO patients [14]. A limited number of studies focused on the clinical outcome of patients with CN-NVO [11]. Moreover, previous research mentioned an increased incidence of patients with CN-NVO who did not respond to empirical antimicrobial treatment [15].

The current narrative review will provide an overview of the definition, diagnosis, and management of CN-NVO.

## 2. Information Sources and Search Strategies

A comprehensive search across multiple databases was conducted on 5 March 2024. The databases reviewed included Scopus via Elsevier (1970+); Ovid MEDLINE^®^ (1946+), which encompasses epub ahead-of-print, in-process, and non-indexed citations; and Ovid MEDLINE^®^ Daily. Additionally, the EBM Reviews—Cochrane Central Register of Controlled Trials (January 2024), the EBM Reviews—Cochrane Database of Systematic Reviews (2005+), and Ovid Embase (1974+) were thoroughly searched.

The search strategies were developed and executed by an experienced medical librarian, with input from the study investigators. The process employed controlled vocabulary combined with relevant keywords, including but not limited to “Discitis”, or “Spondylitis”, or “Diskitis” or “Discitis”, “Discospondylitis” or “Diskospondylitis”, “Osteodiscitis”, “Spondylodiscitis” or “Spondylodiskitis”, “Osteomyelitis”, or “Spine-Infection” and “culture-negative”.

## 3. Definitions

Uniform diagnostic criteria for NVO are lacking [16]. Traditionally, they consisted of a variegate combination of clinical features consistent with NVO and the presence of spinal structural involvement on conventional radiologic images, including radiographs, computed tomography (CT) scans, nuclear medicine (combined In and Ga scans, and PET/CT), or magnetic resonance imaging (MRI) [5,6,7,8,9,10,11]. Conventional microbiology based on blood cultures, serology, cultures, or molecular methods applied to samples obtained invasively from the vertebral bone, disc, or fluid aspiration plays a crucial role in identifying the causative agent. Additionally, histopathology and cytology are increasingly attracting attention to expand the diagnostic landscape [17,18].

Even less consensus exists on the CN-NVO definition across the literature (Supplementary Materials Table S1). CN-NVO traditionally refers to a syndrome featuring clinical characteristics and radiological evidence consistent with NVO without any specific microbe being detected in the patient’s blood culture or biopsy sample [11,15].

## 4. Identifying the Drivers of Culture-Negative Outcomes in NVO

### 4.1. CN-NVO Due to Prior Exposure to Antimicrobials

Administering antimicrobial therapy before a definitive etiological diagnosis is established in these patients can clear the bloodstream infection, if present, or reduce the bacterial burden of disc space/bone tissue and jeopardize the sensitivity of standard culture techniques. Therefore, in all but critically ill patients with signs of sepsis or with neurological compromise that requires surgery, it is suggested to withhold empiric antimicrobials [19] and perform a watchful waiting approach, for which it has already been shown that an antimicrobial-free period of at least four days can maximize the yield of the invasive diagnostic procedure [20]. In a systematic review and meta-analysis by McNamara et al., the biopsy in patients who had previously taken antibiotics resulted in a yield of 32%, which was not significantly different from the group who did not take antibiotics (43%) [21]. However, a recent study by Maamari et al. revealed that the yield of image-guided biopsy could be reduced by threefold due to prior antibiotic use [20]. Moreover, another study by Avenel et al. stated that prior antibiotic administration is associated with a 2.31 times higher negative culture [22].

### 4.2. CN-NVO Due to Fastidious or Difficult-to-Cultivate Pathogens

Numerous human pathogens, particularly zoonotic or vector-borne bacteria, such as Brucella, *Burkholderia pseudomallei*, *Mycoplasma* spp., *Ureaplasma* spp., and *Kingella* spp. (especially in children), as well as rare agents like fungi, parasitic infections, and non-tuberculous mycobacteria, are challenging to cultivate and may cause NVO [23,24,25,26,27,28,29,30]. This challenge is exacerbated in patients with impaired immune systems, where these pathogens exhibit almost total physiological dependence on the host. Consequently, their multiplication rates decrease significantly outside their ideal biological environment [31,32]. Historically, isolating these fastidious pathogens required extended incubation periods; however, advanced automated blood culture techniques have reduced the isolation time for many [33].

Pott’s disease, a vertebral osteomyelitis caused by the *Mycobacterium tuberculosis* complex, exemplifies another fastidious pathogen. Relying solely on positive cultures to diagnose such infections can result in low sensitivity. Thus, additional laboratory reference standards are essential for accurate diagnosis. These standards include the histological identification of caseating granulomas, smear staining to detect acid-fast bacilli (AFB), serological indicators of inflammation, immunological assays, and molecular diagnostic techniques. A definitive diagnosis often necessitates combining the typical clinical presentation with indirect evidence from radiological and laboratory tests [34].

“True” CN-NVO can be explained by infection with intracellular organisms that cannot be cultured in traditional microbiology methods but may be diagnosed with serology or molecular techniques (*Coxiella* and *Bartonella* spp.) [35,36,37]. Interestingly, even *Staphylococcus aureus* can be challenging to cultivate due to its behaviour in osteomyelitis. *S. aureus* can live intracellularly, produce small colony variants (SCVs), invade the osteocyte-lacuna canalicular network (OLCN), and form biofilms and staphylococcal abscess communities, making it difficult to detect using traditional methods [38]. Similar pathogenic mechanisms are noted in other facultative intracellular pathogens like *Pseudomonas aeruginosa* and *Salmonella* spp., warranting further research [39,40]. As pointed out, *Brucella* spp. and the *M. tuberculosis* complex are traditionally discussed separately but require high awareness in nonendemic areas as they may represent culture-negative cases if misdiagnosed.

Rarely can NVO be truly culture-negative if the pathogen is not detectable through traditional culturing. This must be identified through serology or molecular methods because it is an obligate intracellular pathogen. The rarity of this condition underscores the importance of attempting to identify a pathogen through all possible means. Recent attention has been directed toward *Coxiella burnetii* NVO, with a literature review identifying 34 cases of Q fever NVO. This includes 23 patients with complications from adjacent vascular infections and 11 with isolated infections. Diagnosis was often delayed due to the disease’s indolent nature, but PET/CT helped in some cases. Most diagnoses were confirmed by serology and tissue polymerase chain reaction (PCR), with few cases isolating the bacterium through culture [36].

### 4.3. CN-NVO Due to Technical Issues That Can Affect the Yield of Biopsy

Technical issues affecting biopsy yield include an incorrect site sampling, needle gauge, number of samples, and sample type (bone vs. fluid) [41]. An error in sampling can impact the culture’s yield if the specimen is taken from an area with a low microorganism burden.

The main methods for identifying pathogens are image-guided percutaneous needle sampling and open surgical biopsy; the latter yields higher results but requires specialized surgical expertise and may not be widely available [42]. Prior research shows contradictory results regarding the yield of different sample sites, including the disc, the vertebral endplate, and the paravertebral soft tissues [43,44].

Husseini et al. (2021) recommend preferentially aspirating or biopsying fluid samples. If fluid collections are absent, biopsies of paraspinal tissues or the disc are advised, especially when essential structures hinder percutaneous access to the infected bone. Aspiration or core needle biopsy is suggested if imaging shows contiguous infection in adjacent facet joints or posterior paraspinal soft tissues. A biopsy of the affected bone can be performed for unclear imaging needing histopathologic confirmation, with samples sent for microbiologic and pathologic analysis [42].

Another important technical issue is the needle size used for aspiration and/or biopsy. A limited number of previous studies reviewed the impact of needle gauges on the microbiological yield, in which a 20-gauge or larger needle is recommended for aspiration [42,44,45,46].

If a repeat biopsy is necessary, it generally has a lower yield but can still provide positive results, especially in younger patients and those without recent antimicrobial treatment. A second percutaneous needle biopsy should be carried out at least three days after the initial one, as about 75% of infections are detectable after three days of culture [6,42].

Previous research highlights the importance of considering factors like microorganism type, number of culture sets and blood culture bottles, colony-forming units, and growth timing to distinguish pathogens from contaminants [47]. *Cutibacterium acnes* is an organism that might need extended incubation or inoculation of the specimen in blood culture bottles to enhance diagnostic accuracy [48]. However, without clinical, radiological, or histopathological signs of infection, a single culture yielding *C. acnes* from non-instrumented spine sites usually represents contamination [49]. Confirmation from a second spinal aspiration or surgical biopsy is recommended before extended antimicrobial therapy, though heavy *C. acnes* growth might justify immediate treatment, ideally with an antimicrobial covering staphylococcus [50].

### 4.4. Non-Infectious Mimickers of CN-NVO

Clinical and imaging features are classically affected by suboptimal sensitivity. Therefore, differential diagnosis is broad, and current diagnostic methods are increasingly expanding. Recently, there have been advancements in imaging techniques such as ultrasound, dual-energy computed tomography (DECT), MRI, and positron emission tomography (PET)/CT [51].

Previous research has classified NVO mimickers into five distinct categories: degenerative (Modic type I changes, Schmorl’s node and acute intraosseous disc herniation, acute symptomatic calcific discitis), metabolic (such as calcium pyrophosphate dihydrate crystal deposition disease, spinal gout, amyloidosis, destructive spondyloarthropathy of hemodialysis), tumour-related (metastasis or radiation osteonecrosis), inflammatory (sarcoidosis, seropositive spondylitis, SAPHO or spondyloarthritis and Andersson lesions), and miscellaneous (such as pseudoaneurysms, neuropathic arthropathy, or Charcot spine) [2,52,53,54,55]. It is essential to differentiate NVO from these entities because of the substantial treatment and prognostic consequences. In these conditions, it is crucial to emphasize the significance of additional imaging, thorough evaluation of images, and histopathology since they are essential and cannot be underestimated [53]. The “claw sign”, a valuable radiologic finding that can help in distinguishing between a mass originating from a particular organ or structure and one located nearby, which may cause distortion of the organ’s appearance, on diffusion-weighted MRI highly suggests Modic type 1 degenerative changes [54,56]. Moreover, the dominant presence of a solitary end plate increases the probability of degenerative factors, such as Schmorl’s nodes, rather than an infectious etiology. When assessing a possible cause of inflammation, involvement at multiple levels, subluxations, involvement of the posterior components, and the discovery of sacroiliitis would indicate a diagnosis of spondyloarthropathy. Neuropathic arthropathy, called Charcot spine, is frequently misidentified as NVO.

Nevertheless, the diagnosis can be verified by detecting the existence of many bone fragments, specifically on CT scans [2]. Diagnosing spinal gout may be challenging. Patients may exhibit acute crystal-induced inflammation, resulting in severe pain and fever that can resemble infectious discitis or facet joint septic arthritis during clinical examination and imaging studies. Alternatively, patients may experience a gradual onset of pain and neurological symptoms, with tophi being misinterpreted as spinal masses on imaging [57]. However, it is crucial to rule out crystal deposition diseases because incorrectly missing this diagnosis can significantly affect patient outcomes, and prompt treatment can be initiated if the diagnosis is accurately determined. An extensive structured approach to gout is described elsewhere [58].

These categories should not be seen as reciprocally exclusive, as some aetiologies can overlap depending on various factors (e.g., *S. aureus* can produce intracellular as well as extracellular infections, *Brucella* spp. is known to be a fastidious organism, but serology can greatly help in diagnosis, and tubercular infection (Pott’s disease) can be rare in nonendemic countries but highly prevalent in other settings).

## 5. Diagnostic Work-Up

Patients must be evaluated to rule out NVO in the following conditions: (a) new or worsening back or neck pain and fever, (b) new or worsening back or neck pain and elevated erythrocyte sedimentation rate (ESR) or C-reactive protein (CRP), (c) new or worsening back or neck pain and bloodstream infection or infective endocarditis, (d) fever and new neurologic symptoms with or without back pain, and (e) new localized neck or back pain, following a recent episode of *S. aureus* bloodstream infection. A comprehensive neurologic and medical examination should encompass an evaluation of both motor and sensory function, including an assessment for intestinal and urine incontinence and signs and symptoms indicative of infectious endocarditis [59]. The Infectious Diseases Society of America (IDSA) guidelines recommend conducting two sets of bacterial blood cultures (one aerobic bottle and one anaerobic bottle for each set) in individuals suspected to have NVO. Blood cultures are positive in 58% of the patients suffering from NVO and may obviate the necessity of biopsies [59,60]. However, an image-guided aspiration biopsy is necessary for all patients suspected of NVO when a microbiological diagnosis for a recognized related pathogen (such as *S. aureus*, *Staphylococcus lugdunensis*, or *Brucella* spp.) has not been confirmed by blood cultures or serological assays [59]. CT or fluoroscopy is commonly used to guide percutaneous biopsies and aspirations [61]. Percutaneous biopsies, which focus on bone, disc, and nearby infected spinal areas, including facet joints or paraspinal soft tissues, such as abscesses, had microbiologic yields ranging from 30.4% to 91% [2]. If the blood culture and initial biopsy are negative, it is recommended to wait another 48 h before performing the second biopsy (image-guided or open) and before initiating empiric antimicrobial treatment for clinically stable patients [2,62]. It is necessary to send specimens for microbiologic and histopathologic evaluation [2]. Patients with CN-NVO should be evaluated for possible causes such as fungal, zoonotic, parasitic, and mycobacterial infections [2]. Physicians should also rule out endocarditis as a source of infection using transesophageal echocardiography (TEE) among patients with CN-NVO [63].

Another guideline by the University of Michigan indicated that all suspected patients should be evaluated regarding neurologic impairment. Moreover, the authors recommended conducting laboratory analysis, including a complete blood count (CBC), ESR, CRP levels, a basic metabolic panel, urinalysis, urine culture, and two sets of blood cultures. Furthermore, it is recommended to carry out emergent spine imaging, for which MRI is preferred; however, if the MRI is contraindicated, the patient can undergo a CT myelogram or CT with IV contrast. Finally, a biopsy should be considered if the imaging suggests VO, but the blood culture is negative [64].

The French Society of Infectious Diseases recommended a similar diagnostic procedure; however, whole-body 18-fluoro-2-deoxy-D-glucose (FDG) PET/CT with a measurement of the Standardized Uptake Value (SUV) of each focus was recommended in cases of contraindication to MRI. It should be mentioned that the MRI should encompass a complete examination of the whole spine, focusing on the affected level(s), and involve imaging in at least two different planes (sagittal and axial), using sagittal T1-weighted, T2-weighted with fat suppression, and T1-weighted sequences following the administration of gadolinium chelate [65].

Furthermore, infectious diseases specialists appointed by the European Society of Clinical Microbiology and Infectious Diseases (ESCMID) mentioned a similar approach for diagnosing NVO [66].

**Figure 1:**
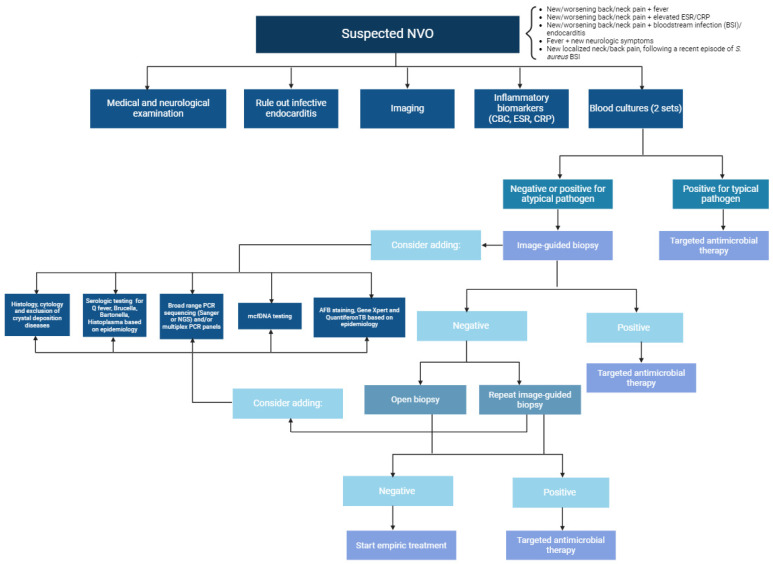
The approach for diagnosis of native vertebral osteomyelitis.

Integrating molecular diagnostics into culture-based methods for diagnosing NVO could enhance diagnostic accuracy. This is particularly important because traditional diagnostics may not detect pathogens, especially in patients receiving antimicrobial treatment or infections caused by fastidious organisms [67]. Compared to culture, the diagnostic sensitivity and specificity of 16S rRNA broad-range PCR were 88.5% and 83.5%, respectively. The PCR detection rate (34.6%) was higher than that of bacterial culture (25.0%) due to the presence of fastidious and uncultivable species in the samples and the administration of antimicrobials to the patients [68]. Previous research illustrated that the use of PCR on the 16S ribosomal RNA (rRNA) gene in suspected patients of NVO has provided evidence of its ability to enhance accuracy and expedite the diagnostic process [2]. Furthermore, another study by Both et al. (2023) showed that the targeted use of the 16S/18S-PCR assay in culture-negative patients may enhance efficiency and the diagnostic yield moderately [67]. Figure 1 illustrates a flowchart for the diagnosis of NVO.

Metagenomic next-generation sequencing (mNGS) is becoming increasingly vital in diagnosing clinical infections. mNGS involves extracting genetic material (DNA or RNA) from clinical samples and sequencing analysis to identify all pathogens present comprehensively. It has shown significant potential and utility in diagnosing complex and rare infectious diseases [69]. mNGS is a powerful sequencing technology that enables the complete sequencing of all microbial DNA in a sample without the requirement of preselection or culturing of specific bacteria [70]. The aforementioned method is an accurate detection technique, allowing physicians to promptly and precisely identify infections, suggest suitable treatment approaches, and contribute to disease monitoring and control [71]. Based on the previous literature, the sensitivity and specificity of mNGS were reported at 95.5% and 31.4%, respectively [72]. The positive rate of mNGS for diagnosing NVO is markedly higher than traditional diagnostic methods. A higher rate of cure can be achieved by administering antimicrobial agents based on the results of mNGS [73]. Classical limitations of mNGS, such as higher costs, the need for complex training for data analysis, stringent contamination control measures, the potential errors due to the host genetic background, and longer turnaround times compared to 16S rRNA PCR coupled with Sanger sequencing, have been improved in recent years [74].

Furthermore, cell-free DNA is a novel noninvasive technique that showed promising results among patients suffering from NVO, periprosthetic joint infections, osteoarticular infections, febrile neutropenia, intravascular infections, and infective endocarditis [75,76]. The aforementioned test may also help physicians track the eradication of infection during the duration of treatment [75]. However, further studies are needed to investigate the use of cell-free DNA, especially in the diagnosis of NVO.

## 6. Clinical Management

The objectives of the treatment for CN-NVO are to eliminate the infection, establish stability in the spinal region, alleviate pain, prevent or reverse neurological deficits, and avoid the reoccurrence of the condition [77]. When the culprit pathogen cannot be isolated, the treating physician must primarily rely on epidemiological patterns, susceptibility rates, and the risk of MDR pathogens to select the most appropriate empiric antimicrobial, having exhausted all other attempts at isolation. Moreover, it is also imperative to consider the capability of bone and disc penetration of the chosen drug [77], the patients’ safety profile, and the ability to endure extended treatment periods, whether orally or intravenously.

The predominant pathogen responsible for culture-positive NVO is methicillin-sensitive *Staphylococcus aureus* (MSSA) [13]. In elderly patients, the incidence of *S. aureus* infections is relatively low, whereas the incidence of Gram-negative bacterial infections and enterococcal infections is relatively high [78]. Moreover, the literature underscores that antimicrobials that are effective against methicillin-resistant *Staphylococcus aureus* (MRSA) should be added to treatment strategies based on local epidemiological data [77]. The effect of the chosen empirical antimicrobial needs to be monitored depending on important indicators such as fever, physical examination, and changes in inflammatory markers, including white blood cell count, ESR, and CRP levels.

Several therapeutic agents were recommended based on previous research, including beta-lactam/beta-lactamase inhibitors, cephalosporins, carbapenems, fluoroquinolones, glycopeptides, glycylcycline, macrolides, and oxazolidinones. The aforementioned antimicrobial agents were utilized solely or in combination to treat CN-NVO. A comprehensive assessment and algorithmic proposal appear difficult due to the literature’s sparse data of low-quality evidence.

A guideline by the University of Michigan suggested administering vancomycin plus ceftriaxone for the empirical treatment of vertebral osteomyelitis [64]. Gatti et al.’s recent multidisciplinary opinion article suggested administering levofloxacin and rifampicin for CN-NVO patients with a low risk of multidrug resistance (MDR). Daptomycin + fosfomycin or ceftobiprole should be used for patients who are at high risk for MRSA or methicillin-resistant *Staphylococcus epidermidis* (MRSE), which can be changed to oral minocycline + rifampicin or dalbavancin + rifampicin at the time of discharge. The authors further recommended adding ertapenem to the previously mentioned antibiotics for patients at high risk for MRSA/MRSE and extended-spectrum beta-lactamase (ESBL) Gram-negative bacteria [79].

According to the French Society of Infectious Diseases, intravenous antimicrobials are inferior to oral antimicrobials. Oral antimicrobials with high bioavailability, including fluoroquinolones, clindamycin, and rifampin, can be utilized for early switch. Moreover, dalbavancin is a recently introduced long-acting antimicrobial that can be administered to patients with CN-NVO suspected of being infected with Gram-positive bacteria [65].

According to the 2015 IDSA guidelines for NVO [59], physicians should consider regimens that cover *Staphylococci*, including MRSA, *Streptococci*, and Gram-negative bacilli. Examples of such regimens could involve vancomycin paired with a third- or fourth-generation cephalosporin. If allergies or intolerance is present, alternatives may include daptomycin and a quinolone. Empiric anti-anaerobic, antifungal, and antimycobacterial therapies are generally not recommended unless specific indications exist. Various regimens were proposed, potentially including combinations like vancomycin with ciprofloxacin, vancomycin with cefepime, or vancomycin with a carbapenem. A description of different types of antimicrobial agents mentioned in previous studies can be found in Table S2 (Supplementary Materials).

## 7. Duration of Treatment

The duration of antimicrobial therapy for NVO generally spans six to twelve weeks, utilizing either intravenous or bioavailable oral antimicrobial drugs, as per IDSA guidelines and extrapolating data from an OVIVA trial from the setting of general bone and joint infections [59,80]. Moreover, other studies recommended at least six weeks of antimicrobial treatment [81,82]. Based on a systematic review and meta-analysis by Passerini et al. (2022), the existing evidence is inadequate in determining whether there is a difference in the proportion of patients experiencing failure or relapse among patients with an early switch to oral antimicrobial therapy (<2 weeks) compared to a non-early switch in bacterial NVO. Further high-quality research is required before early transition can be routinely advised [83].

Previous research indicated that the duration of treatment for a culture-negative group was shorter than that for a culture-positive group [10,11]. However, there is insufficient evidence to recommend an antimicrobial regimen lasting less than six weeks.

Previous studies demonstrated that an extended period of antimicrobial administration should be applied for patients with risk factors, including age > 75 years, MRSA infection, immunosuppression, diabetes mellitus, end-stage renal disease (ESRD), endocarditis, or neurological deficit [81,84].

## 8. Monitoring

It is generally advised that the duration of treatment should be guided by the resolution of the symptoms and the normalization of inflammatory parameters, including ESR and CRP [85]. It is recommended that inflammatory parameters are regularly assessed and clinical evaluations are performed weekly to determine the effectiveness or failure of the treatment. A weekly decrease of 50% in CRP levels is considered a reliable indicator of improvement [86,87]. Conversely, Zarghooni et al. recommend continuing antimicrobial treatment until six consecutive weeks have passed, conditional to inflammatory parameters remaining within the normal range [88].

Follow-up MRI is not recommended as a standard procedure for patients who are clinically responding to treatment [89]. Using follow-up MRI scans with caution is imperative to evaluate the therapeutic response. MRI scans ordered for patients who are clinically responding positively can frequently produce contradictory findings [90]. Repeat MRI is beneficial only in cases with no observed clinical improvement or if conventional radiological imaging reveals signs of worsening [91].

The procalcitonin level has limited power as a marker for diagnosing spondylodiscitis in its early stages. Additionally, it is more expensive to measure than CRP levels and is not useful for monitoring the progress of the condition [92].

## 9. Oral Antimicrobial Therapy

The advantages of oral therapy include a shortened hospital stay, lower incidence of adverse events due to intravascular catheters, and lower costs [83]. When chosen carefully, oral antibiotic therapy was as effective as intravenous therapy in treating bone and joint infections within the first 6 weeks. Discontinuing parenteral antimicrobial or modifying the delivery route may be contemplated depending on the levels of ESR, CRP, and the Visual Analogue Scale (VAS) score for back pain [1,10]. Oral antimicrobials, particularly quinolones, were prescribed and administered more frequently and for longer durations among patients with CN-NVO [10]. Mohamad et al. recommended a treatment regimen consisting of two weeks of intravenous antimicrobials followed by ten weeks of oral cloxacillin/cephalexin and ciprofloxacin, which revealed a high resolution of the CN-NVO [14]. Previous research mentioned other oral antimicrobials, including amoxicillin-clavulanic acid, ciprofloxacin, rifampicin, linezolid, or clindamycin [1].

## 10. Surgical Interventions

The objective of surgical intervention in patients with NVO is stabilizing the spine while ensuring adequate drainage of the abscess, which, if not properly drained, was found to be a significant risk factor for recurrence [13]. The surgical indications include a lack of response to conservative treatment, worsening neurological dysfunction, structural instability, persistent pain unrelieved by analgesics, an extensive bone disease affecting two neighbouring vertebral bodies, a loss of more than 50% in a single vertebral body, unsuccessful CT-guided biopsy, and the presence of an abscess [14,90,93]. The predominant procedures include laminectomies, debridement, and decompression surgeries [4]. Moreover, bed rest, thromboprophylaxis, pain management, spinal immobilization using a collar or halo for cervical lesions, or thoracolumbar or lumbar-sacral orthosis for thoracic or lumbar lesions and rehabilitation when the lesions are healed are also important supportive care measures to be employed within a multidisciplinary approach [77,90].

**Figure 2:**
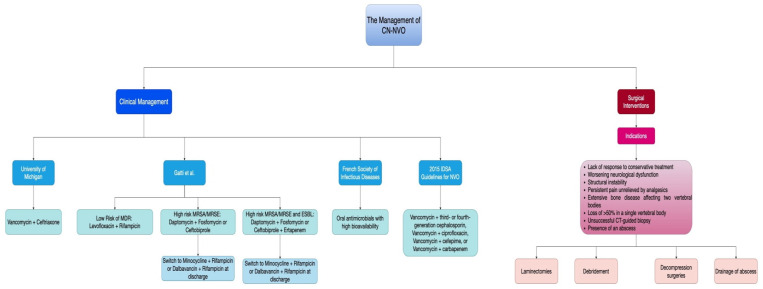
The approach of surgical intervention for the management of culture-negative vertebral osteomyelitis (CN-NVO).

Figure 2 summarizes the clinical and surgical intervention approach for managing CN-NVO based on previous research.

## 11. Outcomes

Kim et al. compared the clinical characteristics and outcomes of 151 patients with CN-NVO and culture-positive NVO (76 vs. 75) and found that the incidence of treatment failure was lower in the CN-NVO group, with a reported rate of 9.2% compared to the group with a positive culture [11].

Based on the literature, culture-negative and culture-positive NVO did not seem to differ in terms of length of stay statistically [10,22,94]. The length of hospitalization among patients with CN-NVO ranges from 18 to 54 days [10,22,94,95]. Dai et al. reported that the length of hospital stay was significantly lower among patients with CN-NVO [95]. The lower incidence of treatment failure and shorter duration of hospital stay may be attributed to certain pathophysiological aspects of CN-NVO, such as a low pathogen burden or absence of an infectious etiology.

Yu et al. reported that recurrence occurred among 3% of the patients [10]. Hopkinson and Patel reported the outcome of six patients with CN-NVO, among which three patients recovered completely; however, two patients did not fully recover the level of mobility prior to the onset of the condition, one patient died, and all had comorbidities, including diabetes and hypertension [94]. Dai et al. reported that 107/126 patients with CN-NVO received surgical intervention and antibacterial treatment, while 19 cases were treated alone with antibacterial medication. Additionally, eight cases experienced a recurrence [95].

Based on Chong et al., the hospital and 1-year mortality rates were both 4% among CN-NVO patients. It is well known that NVO carries a significant burden of sequelae after treatment. Only 14% of the CN-NVO patients recovered completely after discharge. Moreover, back pain remained among 79% of the patients at discharge. The authors also reported that limb weakness and incontinence rates at the time of discharge were 11% and 7%, respectively, among patients with CN-NVO [7].

## 12. Conclusions

Although CN-NVO treatment is challenging, it can be successfully managed with a cooperative partnership among infectious diseases specialists, orthopedics experts, neurosurgeons, pharmacologists, microbiologists, outpatient parenteral antimicrobial therapy (OPAT) teams, rehabilitation experts, and pain management services. Previous studies have identified several antimicrobial agents, each showing different outcomes. This reflects the variability and complexity of this syndrome, particularly when the culprit pathogen cannot be isolated. To mitigate this as much as possible, new diagnostic techniques should be implemented, and traditional methods should be used more effectively. Further research is needed to achieve this, enhance patient care, and advance our understanding of NVO.

## Data Availability

Not applicable.

## Supplementary Materials

The following supporting information can be downloaded at https://www.mdpi.com/article/10.3390/jcm13195802/s1. Table S1: Valuable examples of definitions of culture-negative NVO used across literature, showing lack of consensus and wide variability. Table S2: Antimicrobial treatment for culture-negative native vertebral osteomyelitis (CN-NVO) mentioned in the literature. References [9,11,13,14,15,77,78,94,95,96,97,98,99,100,101,102,103,104,105,106,107,108,109,110,111,112,113,114,115,116,117,118,119,120,121,122,123,124,125,126,127,128,129,130].
