# Supplementary material for: Culture-Negative Native Vertebral Osteomyelitis: A Narrative Review of an Underdescribed Condition
Source: J Clin Med. 2024 Sep 28;13(19):5802. doi: 10.3390/jcm13195802 (PMC11477431; doi:10.3390/jcm13195802)
Supplement: Supplementary file 1 [file jcm-13-05802-s001.zip › jcm-3221645-supplementary.pdf]

Table 1: Valuable examples of definitions of culture-negative NVO used across literature, showing lack of consensus and wide variability

Author	Year	Country	Definition
Bhagat (1)	2007	UK	A combination of back or neck pain, pyrexia, elevated inflammatory markers and imaging findings with a background history of risk factors, previous surgery or septicaemia were considered to establish the diagnosis of discitis.<br>Patients in the study group (Negative culture) met the following criteria: laboratory evidence of inflammation; imaging study evidence of discitis; at least two negative blood cultures; negative percutaneous disco-vertebral biopsy (PDVB) if carried out; negative histology for granulomatous inflammation or fungal infection; negative microbiology after prolonged cultures for mycobacterium tuberculosis; negative microbiology from the surgical specimen if obtained.
Luzzati (2)	2009	Italy	No specific microbiologic or histological diagnosis could be made but suspected spinal infection was confirmed by the favorable response (recovery or qualified recovery) to empirical antimicrobial therapy.
Ziu (3)	2014	USA	Diagnosis of spinal infection was additionally given in cases with suggestive clinical features and appropriate radiological changes, in which microbial cultures remained negative, but the patients had elevated inflammatory markers and/or responded favorably to antimicrobial therapy.
Kim (4)	2014	Korea	Clinically-diagnosed pyogenic vertebral osteomyelitis was defined as a patient with clinical features and radiological findings compatible with PVO but without an identified microorganism.
Weissman (5)	2014	USA	Cases without microbiology results were included and considered culture-negative if the clinical syndrome, radiological findings and/or histopathology was consistent with vertebral osteomyelitis.
Kehrer (6)	2015	Denmark	The microbiological etiology was based on having a positive culture or polymerase chain reaction (PCR) with a recognized pathogen from blood, pus, bone, intervertebral disc, or spinal fluid. Remaining cases were defined as having unknown microbiological etiology.
Ribera (7)	2016	Spain	Diagnosis was considered definitive if a pyogenic microorganism was isolated in blood cultures or spinal biopsy specimens, and probable if no organism was isolated. Biopsies whose histology suggested discitis but that contained no granulomas or identifiable organisms were classified as negative.
Kim (8)	2016	Korea	Culture-negative pyogenic spondylitis was diagnosed when all of the following criteria were met: (1) Bacterial culture of spinal

			tissue grew no microorganisms. If blood cultures were performed, they also grew no bacteria. (2) The symptoms and signs of infectious spondylitis improved with empirical antimicrobial ineffective against M. tuberculosis. (3) M. tuberculosis PCR was negative if performed.
Terreaux (9)	2016	France	Biopsies whose histology suggested discitis but that contained no granulomas or identifiable organisms were classified as negative.
Ghobrial (10)	2017	USA	In the presence of sterile cultures, final study inclusion was made by the intraoperative clinical determination of the staff neurosurgeon. This determination was guided by the presence of frankly purulent material, phlegmon, or the setting of frank soft tissue inflammation.
Chang (11)	2018	Taiwan	Culture-negative hematogenous vertebral osteomyelitis (CN-VO) was defined as the patients had compatible clinical signs/symptoms and featured radiologic findings, but no causative agent(s) was isolated.
Martin-Alonso (12)	2018	Spain	The presumptive diagnosis was based on a combination of clinical findings (local pain, fever, neurological defect), laboratory findings (increased ESR and CRP, leukocytosis) and radiological<br>findings (images of disc and/or vertebral involvement compatible with spondylodiscitis in CT and/or spinal MRI studies). On occasions in which a positive culture was not achieved, empirical antimicrobial treatment was started, which was maintained according to the subsequent clinical, analytical and radiological findings.
Ponciano (13)	2018	Portugal	Empirical etiologic diagnosis was based on clinical suspicion, whenever microbiologic findings did not fulfill the definitive criteria but was supported by a favorable response to antimicrobial therapy.
Lee (14)	2019	Korea	The diagnosis of pyogenic spondylodiscitis (PS) was based on clinical features, laboratory and imaging findings, tissue pathological findings, and culture results, which led to the medical decision on antimicrobial therapy and the compatible response. PS was classified as culture-positive or culture-negative according to the results of bacterial cultures obtained using blood and/or tissue specimens.
Vettivel (15)	2019	New Zealand	Osteomyelitis was confirmed by clinical, radiologic, and microbiologic analyses. For culture-negative cases, the diagnosis of vertebral column osteomyelitis (VCO) was made if there was no history of an inflammatory process that could mimic VCO and if there was a positive response to empiric antimicrobial therapy.
Alas (16)	2020	USA	The diagnosis of spondylodiscitis was confirmed by clinical, radiologic, and hematologic studies. For culture-negative cases,

			the diagnosis was made after ruling out other possible processes of inflammation and if there was a positive response to empiric antimicrobial therapy.
Amsilli (17)	2020	France	Vertebral osteomyelitis (VOM) was considered microbiologically documented accord- ing to the following definitions.<br>1. The diagnosis of pyogenic VOM was confirmed in each of the following cases: (a) Positive cultures or positive polymerase chain reaction (PCR) on vertebral biopsies (VB), by computerized tomography (CT)-guided percuta- neous (CtB) or surgical vertebral biopsy (SuB) (b) AND/OR, positive blood cultures (BCs) (c) AND/OR, positive cultures or positive PCR on others significant samples (e.g. psoas abscess, articular or lum- bar punctures).<br>2. The diagnosis of mycobacterial VOM was confirmed by:<br>(a) Positive Ziehl-Neelsen stain, positive cultures on Lowenstein media (b) AND/OR, positive specific PCR for Mycobacterium of the complex tuberculosis on VB (c) AND/OR, other significant samples (e.g. sputum, bronchial aspiration, urine, abscess, lymph node, bone biopsy) (d) AND/OR, pathology evocative of tuberculosis on VB (granulomatosis with caseous necrosis). Patients whose samples did not meet these definitions were considered as non-documented cases.
Park (18)	2021	Korea	In patients with negative culture results, pyogenic spondylitis (PS)was diagnosed when all the following criteria were satisfied: no histologic evidence of granulomatous inflammation or fungal infection; negative results of a prolonged cultures for *Mycobacterium tuberculosis* and the polymerase chain reaction for Mycobacterium complex; and intraoperative findings consistent with pyogenic infection such as purulent discharge.
Yamada (19)	2022	Japan	For culture-negative cases, pyogenic vertebral osteomyelitis (PVO) was diagnosed if the patient had a history of a clinical (fever, back pain, or abnormal inflammatory value of blood examination) and radiological inflammatory process that could be associated with PVO.
Jeon (20)	2023	Korea	Pyogenic spine infection (PSI) with appropriate clinical features and radiological findings, but with no identification of causative bacteria, were defined as culture-negative PSI.

Table 2: Antimicrobial treatment for culture-negative native vertebral osteomyelitis (CN-NVO) mentioned in the literature

Drug Class	Drug Name	Covering Pathogen	Study description
First-generation Cephalosporines	cefazolin, cephalothin, cephapirin, cephradine, cefadroxil, and cephalexin	Active coverage against the majority of gram-positive cocci, including staphylococci spp. and streptococci spp., along with certain gram-negative bacteria.	Several studies recommended first-generation cephalosporines as the empirical treatment for CN-NVO (4,21–23).<br>Kim et al., the rate of treatment failure was 5% in CN-NVO patients who were administered first-generation cephalosporins (4).<br>Lee et al. initially administered first-generation cephalosporins as an empirical therapy for CN-NVO; however, they further utilized antimicrobial for resistant organisms, including antimicrobial for methicillin-resistant isolates (vancomycin, teicoplanin, and linezolid), fourth-generation cephalosporin, and carbapenem. The recurrence and 1-year mortality rates in the study were recorded at 12% and 5%, respectively (21).<br>Guo et al. administered cephalosporines in addition to early surgical intervention for CN-NVO (24).
Third-generation Cephalosporines	ceftriaxone, cefdinir, cefixime, cefditoren, cefpodoxime, ceftazidime, cefoperazone, ceftizoxime, and ceftibuten.	Gram-negative and gram-positive organisms. However, they are more active against gram-negative bacteria and organisms resistant to the first and second generation cephalosporins.	Kim et al. administered third generation cephalosporines for elderly individuals (25).<br>Pola et al. utilized it for individuals who were susceptible to gram-negative infections, including concomitant abdominal, pelvic, and urinary tract infections (26).<br>Third generation cephalosporines was administered for five patients suffering from CN-NVO, of which metronidazole and teicoplanin were added for two patients. However, flucloxacillin used only for one patient (27).
Carbapenems	Imipenem/ cilastatin, meropenem,	Mainly gram-positive	Lebre et al. administered broad-spectrum antimicrobial, specifically carbapenems, to patients with an unclear cause for an

	doripenem, and Ertapenem		average duration of 3.5 months. However, the authors did not provide specific information regarding the outcome of this treatment (28).
B-lactam/b-lactamase	Amoxicillin / clavulanate	Gram-positive / gram-negative	Yemisci et al. (2010) reported a case of CN-NVO who was successfully treated with three months administration of oral amoxicillin and b-lactamase inhibitor (29).
B-lactam + third-generation cephalosporines	Flucloxacillin + ceftriaxone	Gram-positive / gram-negative + Gram-negative and gram-positive organisms. However, they are more active against gram-negative bacteria and organisms resistant to the first and second generation cephalosporins.	Wenk et al. (2022) administered a combination of intravenous flucloxacillin and ceftriaxone for the treatment of 33 patients with CN-NVO, followed by oral clindamycin and ciprofloxacin (30).
B-lactam + fusidane	Cloxacillin + fucidic acid	Gram-positive / gram-negative + narrow-spectrum antimicrobial active against gram-positive bacteria	Menon et al. (2016) administered cloxacillin and fucidic acid for the treatment of CN-NVO patients. However, they used added meropenem, amikacin, amoxicillin / clavulanate, ciprofloxacin, rifampicin, and doxycycline (31).
Oxazolidinone	Linezolide	Gram positive bacteria	Rocha et al. (2015) administered the linezolide for one patient suffering from CN-NVO associated with tunneled cuffed hemodialysis catheters, which resulted in the survival of the patient (32).
Lipoglycopeptide	Dalbavancin	Wide-range of gram-positive bacteria	Streifel et al. (2022) administered dalbavancin for seven patients suffering from CN-NVO. However, the authors did not mention the outcome of the antimicrobial treatment (33).
B-lactam/b-lactamase	Amoxicillin / clavulanate intravenously	Gram-positive / gram-negative	In a study by Luzzati et al. (2009), 18 patients suspected of having a spine infection were treated with a combination

inhibitor + Fluoroquinolone	at a dose of 1-2 g three times a day or orally at a dose of 1 g three times a day + ciprofloxacin 200-400 mg twice daily or orally at 500 mg twice daily		of antimicrobial. The treatment included a b-lactam/b-lactamase inhibitor (specifically, amoxicillin/clavulanate) administered intravenously at a dose of 1-2 g three times a day or orally at a dose of 1 g three times a day for patients with normal kidney function. Additionally, a fluoroquinolone antimicrobial (specifically, ciprofloxacin) was given intravenously at 200-400 mg twice daily or orally at 500 mg twice daily for patients with normal kidney function. The treatment duration was 4±1.5 months, and as a result, 82% of the patients recovered completely (2).
Glycylcycline	Tigecycline	Gram-positive / gram-negative	Based on the literature, tigecycline demonstrated high bone penetration in preclinical animal tests (34). In a retrospective study, Seyman et al. evaluated 15 patients with culture-negative PVO who were treated with tigecycline as a secondary empirical therapy. The initial dose of tigecycline was a 100 mg infusion, followed by two 50 mg infusions daily. This treatment was given for an average duration of 8.3 weeks. The patients had previously not responded to the primary empirical therapy, which consisted of ampicillin-sulbactam alone or combined with ciprofloxacin. The study found that all patients achieved sustained clinical success (35). In addition, several studies have demonstrated the effectiveness of tigecycline in patients with post-neurosurgical PVO who did not respond to various antimicrobial treatments, as well as in cases of osteomyelitis caused by MRSA and other causes (36–38). Nevertheless, the cost of tigecycline, compounded by potential gastrointestinal side effects, including vomiting and nausea, can restrict its usage (22,35).

Fluoroquinolone + Rifampicin	Levofloxacin/ ciprofloxacin + rifampicin	wide range of aerobic gram-positive and gram-negative organisms + mainly gram-positive	A case series conducted by Viale et al. revealed that using levofloxacin and rifampicin as empiric antimicrobial regimens for culture-negative pyogenic spondylodiscitis yields comparable effectiveness to treatments which have been tailored based on culture results for microbiologically approved pyogenic spondylodiscitis (39).<br>In a systematic review, Cordero-Delgado et al. highlighted that fluoroquinolones can be combined with rifampicin to treat pyogenic spondylodiscitis initially. They also recommend adding a vancomycin pharmacological agent to this combination for treating suspected methicillin-resistant strains. However, they do acknowledge that the available information is favorable, even though it is not conclusive (40).<br>Fesatidou et al. reviewed one immunocompromised patient suffering from concurrent CN-NVO and iliopsoas abscess, for whom ciprofloxacin PO and clindamycin for four weeks, followed by ciprofloxacin PO and rifampicin PO for three weeks were administered, and no recurrence was noted (41).
Macrolides and quinolones	ciprofloxacin and clindamycin	staphylococcal and Gram-negative bacteria	Another empirical therapy that has been advised in the literature involves the use of a combination of ciprofloxacin and clindamycin. This combination offers activity against both staphylococcal and Gram-negative bacteria, as well as suitable penetration into bones and discs. Additionally, it allows for an early transition to oral treatment (42,43).<br>Fesatidou et al. reviewed one immunocompromised patient suffering from concurrent CN-NVO and iliopsoas abscess, for whom ciprofloxacin PO and clindamycin for four weeks, followed by ciprofloxacin PO and rifampicin PO for three weeks were administered, and no recurrence was noted (41).

Glycopeptide and carbapenems	Vancomycin and imipenem	Gram-positive bacteria, including MRSA and various aerobic and anaerobic gram-positive and gram-negative organisms	Dai et al. reviewed 126 CN-NVO patients. A total of 107 patients received surgical intervention combined with antimicrobial therapy, whereas 19 cases were treated solely with antimicrobial medication. However, recurrence was reported among eight patients (44).
Glycopeptide and fluoroquinolones	Vancomycin and ciprofloxacin/ levofloxacin/ moxifloxacin	Gram-positive bacteria, including MRSA and wide range of aerobic gram-positive and gram-negative organisms	Dai et al. reviewed 126 CN-NVO patients. A total of 107 patients received surgical intervention combined with antimicrobial therapy, whereas 19 cases were treated solely with antimicrobial medication. However, recurrence was reported among eight patients (44). Fesatidou et al. reviewed two immunocompromised patients suffering from concurrent CN-NVO and iliopsoas abscess, for whom vancomycin IV and ciprofloxacin PO for eight weeks were administered, and No recurrence was noted (41).
Fluoroquinolones and third-generation cephalosporin	ciprofloxacin/ levofloxacin/ moxifloxacin and ceftriaxone/ ceftazidime/ cefoperazone ceftizoxime	wide range of aerobic gram-positive and gram-negative organisms and gram-negative and gram-positive organisms. However, they are more active against gram-negative bacteria and organisms resistant to the first and second generation cephalosporins.	Dai et al. reviewed 126 CN-NVO patients. A total of 107 patients received surgical intervention combined with antimicrobial therapy, whereas 19 cases were treated solely with antimicrobial medication. However, recurrence was reported among eight patients (44).

References:

1. Bhagat S, Mathieson C, Jandhyala R, Johnston R. Spondylodiscitis (disc space infection) associated with negative microbiological tests: comparison of outcome of suspected disc space infections to documented non-tuberculous pyogenic discitis. Br J Neurosurg. 2007 Oct;21(5):473–7.
2. Luzzati R, Giacomazzi D, Danzi MC, Tacconi L, Concia E, Vento S. Diagnosis, management and outcome of clinically- suspected spinal infection. J Infect [Internet]. 2009 Apr 1;58(4):259–65. Available from: https://doi.org/10.1016/j.jinf.2009.02.006
3. Ziu M, Dengler B, Cordell D, Bartanusz V. Diagnosis and management of primary pyogenic spinal infections in intravenous recreational drug users. Neurosurg Focus. 2014 Aug;37(2):E3.
4. Kim J, Kim Y-S, Peck KR, Kim E-S, Cho SY, Ha YE, et al. Outcome of culture-negative pyogenic vertebral osteomyelitis: comparison with microbiologically confirmed pyogenic vertebral osteomyelitis. Semin Arthritis Rheum. 2014 Oct;44(2):246–52.
5. Weissman S, Parker RD, Siddiqui W, Dykema S, Horvath J. Vertebral osteomyelitis: retrospective review of 11 years of experience. Scand J Infect Dis. 2014 Mar;46(3):193–9.
6. Kehrer M, Pedersen C, Jensen TG, Hallas J, Lassen AT. Increased short- and long-term mortality among patients with infectious spondylodiscitis compared with a reference population. Spine J. 2015 Jun;15(6):1233–40.
7. Ribera A, Labori M, Hernández J, Lora-Tamayo J, González-Cañas L, Font F, et al. Risk factors and prognosis of vertebral compressive fracture in pyogenic vertebral osteomyelitis. Infection. 2016 Feb;44(1):29–37.
8. Kim C-J, Kim EJ, Song K-H, Choe PG, Park WB, Bang JH, et al. Comparison of characteristics of culture-negative pyogenic spondylitis and tuberculous spondylitis: a retrospective study. BMC Infect Dis. 2016 Oct;16(1):560.
9. Terreaux W, Geoffroy M, Ohl X, Job L, Cart P, Eschard J-P, et al. Diagnostic contribution of a second percutaneous needle biopsy in patients with spontaneous diskitis and negative blood cultures and first biopsy. Jt bone spine. 2016 Dec;83(6):715–9.
10. Ghobrial GM, Franco D, Theofanis T, Margiotta PJ, Andrews E, Wilson JR, et al. Cervical Spondylodiscitis: Presentation, Timing, and Surgical Management in 59 Patients. World Neurosurg. 2017 Jul;103:664–70.
11. Chang W-S, Ho M-W, Lin P-C, Ho C-M, Chou C-H, Lu M-C, et al. Clinical characteristics, treatments, and outcomes of hematogenous pyogenic vertebral osteomyelitis, 12-year experience from a tertiary hospital in central Taiwan. J Microbiol Immunol Infect. 2018 Apr;51(2):235–42.
12. Martín-Alonso J, Delgado-López PD, Castilla-Díez JM, Martín-Velasco V, Galacho-Harriero AM, Ortega-Cubero S, et al. [Role of surgery in spontaneous spondylodiscitis: Experience in 83 consecutive patients]. Neurocir (English Ed. 2018;29(2):64–78.
13. Ponciano A, Cruz G, Ventura C, Rabadão E, Saraiva da Cunha J. Infectious spondylodiscitis: 5-year analysis of a tertiary hospital in Portugal. Vol. 50, Infectious diseases (London, England). England; 2018. p. 637–9.
14. Lee YD, Jeon YH, Kim YH, Ha KY, Hur JW, Ryu KS, et al. Clinical Characteristics and Outcomes of Patients with Culture-Negative Pyogenic Spondylitis according to Empiric Glycopeptide Use. Infect Chemother. 2019 Sep;51(3):274–83.
15. Vettivel J, Bortz C, Passias PG, Baker JF. Pyogenic Vertebral Column Osteomyelitis in Adults: Analysis of Risk Factors for 30-Day and 1-Year Mortality in a Single Center Cohort

Study. Asian Spine J. 2019 Aug;13(4):608–14.
16. Alas H, Fernando H, Baker JF, Brown AE, Bortz C, Naessig S, et al. Comparative outcomes of operative relative to medical management of spondylodiscitis accounting for frailty status at presentation. J Clin Neurosci Off J Neurosurg Soc Australas. 2020 May;75:134–8.
17. Amsilli M, Epaulard O. How is the microbial diagnosis of bacterial vertebral osteomyelitis performed? An 11-year retrospective study. Eur J Clin Microbiol Infect Dis Off Publ Eur Soc Clin Microbiol. 2020 Nov;39(11):2065–76.
18. Park SC, Chang SY, Gimm G, Mok S, Kim H, Chang B-S, et al. Involvement of L5-S1 level as an independent risk factor for adverse outcomes after surgical treatment of lumbar pyogenic spondylitis: A multivariate analysis. J Orthop Surg (Hong Kong). 2021;29(2):23094990211035570.
19. Yamada K, Ieguchi M, Takahashi S, Nakamura H. Life Expectancy Is Poor in Patients with Diffuse Idiopathic Skeletal Hyperostosis-Related Pyogenic Vertebral Osteomyelitis. Spine Surg Relat Res. 2022 Nov;6(6):654–63.
20. Jeon S, Yu D, Bae SW, Kim SW, Jeon I. Analysis of Clinical Factors Associated with Medical Burden and Functional Status in Pyogenic Spine Infection. J Clin Med [Internet]. 2023;12(7). Available from: https://www.mdpi.com/2077-0383/12/7/2551
21. Lee SH, Kim J, Kim T-H. Treatment Guideline for Patients with Native Culture-negative Pyogenic Vertebral Osteomyelitis. Clin Orthop Relat Res. 2022 Jan;480(1):124–36.
22. Mohamad G, Amritanand R, David KS, Krishnan V, Arockiaraj J. Treatment Strategy and Outcomes in Patients with Hematogenous Culture-Negative Pyogenic Vertebral Osteomyelitis. Asian Spine J. 2019 Feb;13(1):61–7.
23. Yoon SH, Chung SK, Kim K-J, Kim H-J, Jin YJ, Kim H Bin. Pyogenic vertebral osteomyelitis: identification of microorganism and laboratory markers used to predict clinical outcome. Eur spine J Off Publ Eur Spine Soc Eur Spinal Deform Soc Eur Sect Cerv Spine Res Soc. 2010 Apr;19(4):575–82.
24. Guo W, Wang M, Chen G, Chen K-H, Wan Y, Chen B, et al. Early surgery with antibiotic medication was effective and efficient in treating pyogenic spondylodiscitis. BMC Musculoskelet Disord [Internet]. 2021;22(1):288. Available from: https://doi.org/10.1186/s12891-021-04155-2
25. Kim DY, Kim UJ, Yu Y, Kim S-E, Kang S-J, Jun K-I, et al. Microbial Etiology of Pyogenic Vertebral Osteomyelitis According to Patient Characteristics. Open forum Infect Dis. 2020 Jun;7(6):ofaa176.
26. Pola E, Logroscino CA, Gentiempo M, Colangelo D, Mazzotta V, Di Meco E, et al. Medical and surgical treatment of pyogenic spondylodiscitis. Eur Rev Med Pharmacol Sci. 2012 Apr;16 Suppl 2:35–49.
27. Hopkinson N, Patel K. Clinical features of septic discitis in the UK: a retrospective case ascertainment study and review of management recommendations. Rheumatol Int [Internet]. 2016;36(9):1319–26. Available from: https://doi.org/10.1007/s00296-016-3532-1
28. Lebre A, Velez J, Rabadão E, Oliveira J, da Cunha JS, Silvestre AM. Infectious Spondylodiscitis: A Retrospective Study of 140 Patients. Infect Dis Clin Pract [Internet]. 2014;22(4). Available from: https://journals.lww.com/infectdis/fulltext/2014/07000/infectious_spondylodiscitis__a_retrospective_study.10.aspx

29. Yemisci OU, Cosar SNS, Oztop P, Karatas M. Spondylodiscitis Associated With Multiple Level Involvement and Negative Microbiological Tests: An Unusual Case. Spine (Phila Pa 1976) [Internet]. 2010;35(19). Available from: https://journals.lww.com/spinejournal/fulltext/2010/09010/spondylodiscitis_associated_with_multiple_level.33.aspx
30. 17th German Spine Congress, Annual Meeting of the German Spine Society. Eur Spine J [Internet]. 2022;31(11):3163–249. Available from: https://doi.org/10.1007/s00586-022-07413-6
31. Menon KV, Sorour TMM. Epidemiologic and Demographic Attributes of Primary Spondylodiscitis in a Middle Eastern Population Sample. World Neurosurg. 2016 Nov;95:31–9.
32. Rocha A, Castro R, Santos J. Endocarditis and Spondylodiscitis Associated with Tunneled Cuffed Hemodialysis Catheters: Hospitalizations with Poor Outcomes. Int J Artif Organs [Internet]. 2015 Apr 1;38(4):173–7. Available from: https://doi.org/10.5301/ijao.5000401
33. Streifel AC, Strnad L, Sukerman E, Makadia JT, Lewis JS, Douglass AH, et al. 975. Dalbavancin for the Treatment of Vertebral Osteomyelitis. Open Forum Infect Dis [Internet]. 2022 Dec 1;9(Supplement_2):ofac492.817. Available from: https://doi.org/10.1093/ofid/ofac492.817
34. Yin L-Y, Lazzarini L, Li F, Stevens CM, Calhoun JH. Comparative evaluation of tigecycline and vancomycin, with and without rifampicin, in the treatment of methicillin-resistant Staphylococcus aureus experimental osteomyelitis in a rabbit model. J Antimicrob Chemother. 2005 Jun;55(6):995–1002.
35. Seyman D, Berk H, Sepın-Ozen N, Kızılates F, Turk CC, Buyuktuna SA, et al. Successful use of tigecycline for treatment of culture-negative pyogenic vertebral osteomyelitis. Infect Dis (Auckl) [Internet]. 2015 Nov 2;47(11):783–8. Available from: https://doi.org/10.3109/23744235.2015.1062132
36. Sipahi OR, Kahraman H, Mermer S, Pullukcu H, Tasbakan M, Arda B, et al. Tigecycline in the management of post-neurosurgical spondylodiscitis: a review of eight cases. Int J Infect Dis IJID Off Publ Int Soc Infect Dis. 2014 Jun;23:16–9.
37. Kandemir O, Oztuna V, Colak M, Akdag A, Camdeviren H. Comparison of the efficacy of tigecycline and teicoplanin in an experimental methicillin-resistant Staphylococcus aureus osteomyelitis model. J Chemother. 2008 Feb;20(1):53–7.
38. Griffin AT, Harting JA, Christensen DM. Tigecycline in the management of osteomyelitis: a case series from the bone and joint infection (BAJIO) database. Diagn Microbiol Infect Dis. 2013 Nov;77(3):273–7.
39. Viale P, Furlanut M, Scudeller L, Pavan F, Negri C, Crapis M, et al. Treatment of pyogenic (non-tuberculous) spondylodiscitis with tailored high-dose levofloxacin plus rifampicin. Int J Antimicrob Agents. 2009 Apr;33(4):379–82.
40. Cordero-Delgado DA, Moheno-Gallardo AJ, Torres-González R, Mata-Hernández A, Elizalde-Martínez E, Pérez-Atanasio JM. [Evidence and recommendation of empirical antimicrobial treatment in pyogenic spondylodiscitis: systematic review]. Rev Med Inst Mex Seguro Soc. 2017;55 Suppl 1:S6–13.
41. Fesatidou V, Petsatodis E, Kitridis D, Givissis P, Samoladas E. Minimally invasive outpatient management of iliopsoas muscle abscess in complicated spondylodiscitis. World J Orthop. 2022 Apr;13(4):381–7.
42. Cottle L, Riordan T. Infectious spondylodiscitis. J Infect. 2008 Jun;56(6):401–12.

43. Kwon J-W, Hyun S-J, Han S-H, Kim K-J, Jahng T-A. Pyogenic Vertebral Osteomyelitis: Clinical Features, Diagnosis, and Treatment. Korean J Spine. 2017 Jun;14(2):27–34.
44. Dai G, Li S, Yin C, Sun Y, Hou J, Luan L, et al. Culture-negative versus culture-positive in pyogenic spondylitis and analysis of risk factors for relapse. Br J Neurosurg. 2021 Mar;1–5.
